# Determinants of early initiation of breastfeeding in Ethiopia: a population-based study using the 2016 demographic and health survey data

**DOI:** 10.1186/s12884-019-2211-0

**Published:** 2019-02-13

**Authors:** James Rufus John, Sabuj Kanti Mistry, Getahun Kebede, Narendar Manohar, Amit Arora

**Affiliations:** 1 School of Nursing and Midwifery, Western Sydney University, Campbelltown, NSW Australia; 2 Capital Markets Cooperative Research Centre, Sydney, Australia; 3 BRAC James P Grant School of Public Health, BRAC University, Dhaka, Bangladesh; 4 University of Tasmania, Faculty of Health, School of Health Sciences, Launceston, Tasmania Australia; 5 University of Gondar, College of Medicine and Health Sciences, Institute of Public Health, Gondar, Ethiopia; 6 School of Science and Health, Western Sydney University, Campbelltown, NSW Australia; 7 Translational Health Research Institute, Western Sydney University, Campbelltown, NSW Australia; 8 Oral Health Services and Sydney Dental Hospital, Sydney Local Health District, Surry Hills, NSW Australia; 9 Discipline of Child and Adolescent Health, Sydney Medical School, Faculty of Medicine and Health, The University of Sydney, Westmead, NSW Australia

**Keywords:** Timely initiation, Ethiopia, Breastfeeding, Mothers, Infants

## Abstract

**Background:**

Timely breastfeeding initiation is a simple but important measure that has protective effects on infants and mothers. This study aims to determine the predictors of early breastfeeding initiation among mothers residing in Ethiopia.

**Methods:**

This study employed the 2016 Ethiopian Demographic and Health Survey data. A total of 5546 children born during the last 24 months at the time of survey were included for analysis from nine regional states and two city administration areas. Socio-demographic and socio-economic factors including individual, household and community-level factors were examined of their significance against the outcome variable of early initiation of breastfeeding using a mixed-effect logistic regression model.

**Results:**

The proportion of infants who had timely initiation of breastfeeding was 74.3% (*n* = 3064). In the multivariate logistic regression analysis, mothers who delivered with assistance of one or more health professionals had 68% (AOR 1.68; 95% CI: 1.23, 2.29) higher odds of initiating timely breastfeeding. In addition, mothers delivering by a caesarean section had 86% reduced odds of early breastfeeding initiation (AOR 0.14; 95% CI: 0.09, 0.22) when compared to mothers who had vaginal delivery. In terms of socio-demographic factors, the odds of early breastfeeding initiation were more than two and half times higher particularly for mothers residing particularly in Oromiya (AOR 2.58; 95% CI: 1.84, 3.63) and Southern Nations Nationalities and Peoples (SNNP) (AOR 2.75; 95% CI: 1.86, 4.05). In addition, timely breastfeeding initiation was also significantly associated with wealth index with wealthier mothers having 43% higher odds compared to mothers of poorest households (AOR 1.43; 95% CI: 1.07, 1.92). Other factors such as age, gender and birth order of the infant also had significant associations with early breastfeeding initiation.

**Conclusion:**

Early breastfeeding initiation in Ethiopia is inextricably associated with various socio-demographic, biomedical, and socio-economic factors. The study findings can potentially inform mothers and the wider community on the benefits of timely breastfeeding initiation and policymakers and community leaders to target health promotional interventions and resources where needed.

## Introduction

Breastfeeding is an essential primary health care practice for optimal care of a newborn infant [1]. Early initiation of breastfeeding (EIBF) is referred to as feeding through mother’s breast milk to newborn infants within first hour of birth [2]. EIBF has profound implications for both infants and mothers in terms of nutritional, developmental and immunological outcomes [3]. Practice of EIBF enables provision of immunoglobulin and other essential bioactive molecule-rich colostrum for newborn infants that are critical for their immunity, growth and development [4]. There is sound evidence indicating that EIBF is strongly associated with reduced mortality among neonatal and newborn infants [5]. Furthermore, research indicates that EIBF promotes “Maternal-Infant Bonding” resulting in beneficiary outcomes for child’s development [6]. EIBF practice is also proven to have both short and long-term benefits to mothers as it reduces postpartum haemorrhage, lowers risk of obesity post-delivery, improve birth spacing period and reduces the risk of breast and ovarian cancer in the long run [1, 7, 8].

While the health benefits of EIBF are well-recognised, a significant percentage of newborns infants continue not to be breastfed within the first hour as per the World Health Organization (WHO) recommendation. Research indicates that the average prevalence of EIBF is 64% in 128 countries, with one-half of the countries having prevalence of less than 50% [9]. Ethiopia is one of the countries that have implemented several initiatives for improving access and availability of primary care services particularly after the launch of the Health Promotion Program in 2003. Evidence shows a predominant increase in the number of health workforce particularly in rural regions of Ethiopia to promote basic health awareness and intervention [10]. The Ethiopian government has recognized the importance of EIBF and other nutrition programs through National reforms and strategies on infant and young child feeding practices [11]. Recent Demographic Health Survey (DHS) report shows that 74.3% of newborn infants were breastfeed within first hour of birth and infant mortality was estimated to be 48 deaths per 1000 live births [12].

Despite improvements in the prevalence of EIBF from 52% in 2011 to 74.3% in 2016, this percentage still falls well short of the EIBF target of 92% set by the Ethiopian Ministry of Health’s Health Sector Development Program Four (HSDP-IV) [13]. This indicates the presence of existing barriers or new barriers that necessitates further investigations. In addition to this knowledge gap, although there are literatures reporting several drivers and barriers of EIBF across different communities in Ethiopia, most studies are small scale [14–19] or have not used the appropriate definition of timely initiation of breastfeeding as recommended by the WHO [20].

Therefore, there is a strong need of more comprehensive understanding of the current underlying factors of EIBF among mothers of infants under the age of 24 months on a nationally representative sample in Ethiopia. Therefore, this study aims to use the 2016 Ethiopian Demographic and Health Survey (EDHS) to determine individual, household and community level factors that predict EIBF in mothers of infants aged less than 24 months.

## Methods

### Data source, sampling and data collection

The data for this study was extracted from the 2016 EDHS. The 2016 EDHS is the fourth and most recent in the Demographic and Health Survey series in Ethiopia [12]. The survey was conducted in nine regional states and two city administrations of Ethiopia [12]. Further details on sampling strategy can be found in the DHS manual [12].

A total of 16,583 eligible women between 15 and 49 years were approached to be interviewed. A response rate of 95% was observed with 15,683 women completing the interviews. This included both women who were permanent household residents as well as visitors of the household. The interviews included several standard questionnaires recording information ranging from basic socio-demographic information to detailed bio-medical information. Our analysis only included children less than 24 months of age, living with an eligible respondent, in accordance with the denominator of the EIBF definition [2], which resulted in a total weighted sample of 5546.

### Outcome variable

We used EIBF as the outcome variable using the recommended definition as children less than 24 months of age who were breastfed within first hour of birth [2]. This indicator was self-reported by mothers.

### Co-variates

The co-variates included individual, household, and community level actors. Individual factors included socio-demographic characteristics such as mother’s highest education level (categorised as “no education”, “primary”, “secondary or above”) and employment status in the past 12 months (“not working” or “working”), partner’s highest educational level (“no education”, “primary”, “secondary or above”) and employment status (“not working”, “non-agriculture” and “agriculture”), marital status (“never married”, “currently married”, “formerly married”), mother’s age (“15–24 years”, “25–34 years”, “35–49 years”), child’s age (“0–5 months”, “6–11 months”, “12–17 months”, “18–23 months”), and mothers exposure to mass media (“no” or “yes”).

Bio-medical factors included the number of antenatal clinic visits (categorised as “0 visits”, “1–3 visits”, “4 or more visits”), place of delivery (“home” or “health facility”) and mode of delivery (“caesarean section” or “vaginal”), type of delivery assistance (“health professional”, “Traditional birth attendants”, “relatives/untrained workers”, “none”) and mother’s body mass index (BMI) measured by measured by weight (kg)/height (m^2^).

Household factors included household wealth index (categorised as “poorest”, “poorer”, “middle”, “richer” and “richest”), The household wealth index was calculated using scores based on household assets with analyses conducted by the National Population Commission and Inner City Fund (ICF) International based on a methodology developed from previous DHSs [21, 22] and using methods recommended by the World Bank Poverty Network and United Nations International Children’s Emergency Fund (UNICEF) [23].

Community level factors recorded were the place of residence (“rural” or “urban”) and geographical region. The geographical regions were grouped into nine regional states of Ethiopia; namely Afar, Amhara, Benishangul-Gumuz, Gambella, Harari, Oromia, Somali, Southern Nations Nationalities and Peoples’ Region (SNNP), and Tigray***,*** and two city administrations named Addis Ababa and Dire Dawa [12].

### Statistical analysis

Sampling weights were applied for all analyses to manage sampling error and for non-responses. Further details on sample weights can be found in the EDHS report [24].

Descriptive statistics were employed to show the distribution of background characteristics. Since, the survey data were nested in nature with variations among clusters (enumeration areas), we used mixed-effect logistic regression model to determine the true association between timely initiation of breastfeeding and different factors. We considered determinants as fixed effect and cluster variation as random effect. The parameters of the model were estimated through generalised estimating equation (GEE) approach by considering exchangeable correlation structure among clusters [25]. The full model was run with those variables showing *P* < 0.25 in the unadjusted analysis [26]. Meanwhile, the final model was reduced using the backward stepwise procedure and all the variables in the final model were variables for which *P* ≤ 0.05. Both unadjusted and adjusted odds ratios (ORs) were reported with 95% confidence intervals (95% CI). All analyses were performed using statistical software Stata (Version 13.0).

### Ethics approval

This study is a secondary analysis of publicly available dataset where permission was obtained through registering with the DHS website and therefore no ethics approval was required.

## Results

### Baseline characteristics

The prevalence of EIBF in Ethiopia was observed to be 74.3% (*n* = 3064). As summarised in Table 1, a predominant percentage of the children lived in rural areas (87.9%), especially in the regions of Oromiya (44.7%) and SNNP (20%). 59% of mothers reported not working in the past 12 months at the time of survey, and 61% did not have any formal education. In addition to education status, around 72% of mothers reported having poor literacy skills and could not read or write a sentence.

**Table 1:** Individual, household and community level characteristics of children < 24 months of age, Ethiopia 2016

Characteristics	Overall	Initiation of breastfeeding	
	(*n* = 4121)	Within first hour (*n* = 3064)	After first hour (*n* = 1057)
Individual level factors	*N* (%)	*N* (%)	*N* (%)
Socio-demographic factors			
Maternal working status (past 12 months)			
Non-working	2427 (58.9)	1810 (59.1)	617 (58.3)
Working	1694 (41.1)	1254 (40.9)	440 (41.7)
Maternal education			
No education	2487 (60.4)	1851(60.4)	636 (60.2)
Primary	1273 (30.9)	955 (31.2)	318 (30.1)
Secondary and above	361 (8.7)	258 (8.5)	103 (9.6)
Partner’s occupation			
Not working	285 (7.3)	216 (7.4)	69 (7.0)
Non-agriculture	1092 (27.8)	815 (27.7)	277 (28.0)
Agriculture	2548 (64.9)	1905 (64.9)	643 (65.0)
Partner’s education			
No education	1755 (45.0)	1285 (44.0)	470 (48.1)
Primary	1577 (40.4)	1221 (41.8)	356 (36.4)
Secondary and above	568 (14.6)	416 (14.2)	152 (15.5)
Mother’s age (years)			
15–24	1205 (29.2)	875 (28.6)	330 (31.2)
25–34	2097 (50.9)	1608 (52.5)	489 (46.3)
35–49	820 (19.9)	582 (19.0)	238 (22.5)
Marital status			
Never married	76 (1.8)	53 (1.7)	23 (2.1)
Currently married	3879 (94.1)	2898 (94.6)	981 (92.8)
Formerly married	166 (4.0)	113 (3.7)	53 (5.1)
Child’s age (months)			
0–5	1178 (28.6)	841 (27.5)	337 (31.9)
6–11	1052 (25.5)	784 (25.6)	268 (25.4)
12–17	1084 (26.3)	833 (27.2)	251 (23.8)
18–23	808 (19.6)	607 (19.8)	201 (19.0)
Mother’s literacy			
Cannot read at all	2955 (71.7)	2207 (72.0)	748 (70.8)
Can read part/whole sentence	1166 (28.3)	857 (28.0)	309 (29.2)
Mother’s frequency of reading newspaper			
No	3841 (93.2)	2868 (93.6)	973 (92.0)
Yes	281 (6.8)	197 (6.4)	84 (8.0)
Mother’s frequency of listening to the radio			
No	3000 (72.8)	2231 (72.8)	769 (72.8)
Yes	1121 (27.2)	833 (27.2)	288 (27.2)
Mother’s frequency of watching TV			
No	3000 (72.8)	2231 (72.8)	769 (72.8)
Yes	1121 (27.2)	833 (27.2)	288 (27.2)
Bio-medical factors			
Place of delivery			
Home	2536 (61.5)	1884 (61.5)	652 (61.6)
Health facility	1585 (38.5)	1180 (38.5)	405 (38.4)
Mode of delivery			
Non-caesarean	4012 (97.4)	3020 (98.6)	992 (93.8)
Caesarean section	109 (2.6)	44 (1.4)	65 (6.2)
Type of delivery assistance			
Health professional	1539 (37.3)	1180 (38.5)	359 (34.0)
Traditional birth attendant	1400 (34.0)	1069 (34.8)	331 (31.3)
Relatives/untrained workers	560 (13.6)	355 (11.6)	205 (19.4)
None	622 (15.1)	460 (15.1)	162 (15.3)
Antenatal care visits			
None	1425 (34.6)	1080 (35.3)	345 (32.7)
1–3 visits	1313 (31.9)	963 (31.4)	350 (33.1)
4 or more	1383 (33.5)	1021 (33.3)	362 (34.2)
Mother’s BMI (kg/m^2^)			
≤18.5	828 (20.7)	626 (21.0)	202 (19.5)
> 18.5- ≤ 24.9	2908 (72.6)	2157 (72.6)	751 (72.6)
> 24.9- ≤ 29.9	220 (5.5)	153 (5.1)	67 (6.5)
≥ 30	53 (1.2)	38 (1.3)	15 (1.4)
Household factors			
Wealth index			
Poorest	974 (23.6)	725 (23.7)	249 (23.6)
Poorer	893 (21.7)	674 (22.0)	218 (20.6)
Middle	867 (21.0)	648 (21.2)	219 (20.7)
Richer	746 (18.1)	527 (17.2)	219 (20.7)
Richest	642 (15.6)	490 (16.0)	152 (14.4)
Community-level factors			
Residence			
Urban	498 (12.1)	371 (12.1)	127 (12.0)
Rural	3624 (87.9)	2694 (87.9)	930 (88.0)
Region			
Tigray	304 (7.4)	195 (6.4)	110 (10.4)
Affar	40 (1.0)	17 (0.6)	23 (2.2)
Amhara	757 (18.4)	508 (16.6)	249 (23.6)
Oromiya	1841 (44.7)	1433 (46.7)	409 (38.7)
Somali	169 (4.1)	133 (4.4)	35 (3.4)
Benishangul-Gumuz	44 (1.1)	32 (1.0)	12 (1.2)
SNNP	825 (20.0)	644 (21.0)	181 (17.1)
Gamabela	10 (0.2)	6 (0.2)	3 (0.3)
Harari	10 (0.2)	9 (0.3)	1 (0.1)
Addis ababa	105 (2.6)	72 (2.4)	33 (3.1)
Dire dawa	17 (0.3)	16 (0.5)	1 (0.1)

In terms of mothers’ age, overall 51% of mothers were between 25 and 34 years of age at the time of having their first child. Most mothers (95%) reported as currently married at the time of the survey. Of the total births, only 38.5% took place at a health care facility with very low percentage of deliveries by caesarean section (2.6%). In terms of the number of antenatal visits, about 32% of mothers reported to have made at least 1–3 antenatal clinic visits and 33.5% had made more than 4 visits during pregnancy.

### Predictors of EIBF

Of the total sample of 5546 children born in the last 24 months at the time of survey, 73.4% (*n* = 3064) of infants were breastfed within first hour of birth. Table 2 shows unadjusted and adjusted odds ratios (AOR) that were calculated to determine the strength of association between the co-variates and EIBF. As expected, mothers who delivered with assistance of one or more health professionals had 68% (AOR 1.68; 95% CI: 1.23,2.29) higher odds of initiating timely breastfeeding. In addition, type of delivery had significant association with mothers delivering by caesarean section having 86% reduced odds of early breastfeeding initiation (AOR 0.14; 95% CI: 0.09,0.22) compared to mothers who had vaginal delivery.

**Table 2:** Unadjusted and adjusted Odds Ratio for early initiation of breastfeeding in Ethiopia 2016

Characteristics	Unadjusted odds ratio			Adjusted odds ratio		95% CI
	OR	*p*-value	95% CI	OR	*p*-value	
Individual-level factors						
Maternal working status (past 12 months)						
Non-working	1.00			Not retained in the final model		
Working	1.16	0.067	0.99–1.37			
Maternal education						
No education	1.00			Not retained in the final model		
Primary	1.10	0.279	0.93–1.29			
Secondary and above	1.15	0.224	0.92–1.44			
Partner’s occupation						
Not working	1.00			Not taken in the model		
Non-agriculture	1.00	0.980	0.75–1.32			
Agriculture	0.91	0.479	0.70–1.18			
Partner’s education						
No education	1.00			Not retained in the final model		
Primary	1.16	0.091	0.98–1.37			
Secondary and above	1.11	0.289	0.91–1.36			
Mother’s age (years)						
15–24	1.00			Not retained in the final model		
25–34	1.16	0.046	1.00–1.35			
35–49	1.12	0.264	0.92–1.36			
Marital status						
Never married	1.00			Not taken in the model		
Currently married	0.98	0.930	0.60–1.58			
Formerly married	0.75	0.337	0.42–1.35			
Birth order						
First-born	1.00					
Second to fourth	1.31	0.004	1.09–1.57	1.38	0.002	1.13–1.69
Fifth or more	1.31	0.004	1.09–1.59	1.44	0.000	1.18–1.77
Preceding birth interval						
No pervious birth	1.00			Not taken in the model		
< 24 months	1.34	0.012	1.07–1.69			
> = 24 months	1.30	0.003	1.09–1.54			
Mother’s literacy						
Cannot read at all	1.00			Not taken in the model		
Can read part/whole sentence	1.02	0.850	0.86–1.20			
Mother’s frequency of reading newspaper						
No	1.00			Not taken in the model		
Yes	0.98	0.885	0.76–1.27			
Mother’s frequency of listening to the radio						
No	1.00			Not taken in the model		
Yes	0.97	0.707	0.82–1.14			
Mother’s frequency of watching TV						
No	1.00			Not retained in the final model		
Yes	1.23	0.031	1.02–1.49			
Place of delivery						
Home	1.00			Not retained in the final model		
Health facility	1.28	0.002	1.09–1.50			
Mode of delivery						
Non-caesarean	1.00					
Caesarean section	0.26	0.000	0.18–0.37	0.14	0.000	0.09–0.22
Type of delivery assistance						
None	1.00					
Health professional	1.27	0.083	0.97–1.65	1.68	0.001	1.23–2.29
Traditional birth attendant	0.97	0.845	0.74–1.28	1.22	0.201	0.90–1.64
Relatives/untrained workers	0.94	0.700	0.67–1.31	1.10	0.620	0.76–1.59
Antenatal care visits						
None	1.00			Not taken in the model		
1–3 visits	1.06	0.542	0.88–1.26			
4 or more	1.08	0.405	0.90–1.29			
Mother’s BMI (kg/m^2^)						
≤18.5	1.00			Not taken in the model		
> 18.5- ≤ 24.9	1.02	0.811	0.86–1.20			
> 24.9- ≤ 29.9	1.05	0.722	0.80–1.39			
≥30	1.04	0.879	0.63–1.72			
Gender of the child						
Male	1.00					
Female	1.17	0.019	1.03–1.34	1.17	0.035	1.01–1.34
Child’s age (months)						
0–5	1.00					
6–11	1.21	0.038	1.01–1.44	1.22	0.042	1.01–1.47
12–17	1.12	0.217	0.94–1.34	1.15	0.153	0.95–1.40
18–23	1.23	0.042	1.01–1.51	1.20	0.111	0.96–1.49
Household factors						
Wealth index						
Poorest	1.00					
Poorer	1.26	0.031	1.02–1.56	1.01	0.967	0.79–1.27
Middle	1.33	0.022	1.04–1.69	1.04	0.745	0.80–1.36
Richer	1.26	0.071	0.98–1.62	0.96	0.743	0.73–1.25
Richest	1.64	0.000	1.31–2.06	1.43	0.015	1.07–1.92
Community-level factors						
Residence						
Urban	1.00					
Rural	0.73	0.004	0.59–0.90	0.89	0.594	0.59–1.35
Region						
Tigray	1.00					
Affar	0.44	0.000	0.31–0.63	0.52	0.001	0.36–0.76
Amhara	1.20	0.318	0.84–1.72	1.42	0.062	0.98–2.06
Oromiya	2.08	0.000	1.49–2.89	2.58	0.000	1.84–3.63
Somali	1.91	0.001	1.29–2.85	2.19	0.000	1.46–3.28
Benishangul-Gumuz	1.58	0.021	1.07–2.32	1.87	0.002	1.26–2.76
SNNP	2.15	0.000	1.48–3.12	2.75	0.000	1.86–4.05
Gamabela	1.43	0.073	0.97–2.12	1.68	0.012	1.12–2.52
Harari	5.21	0.000	3.08–8.80	7.05	0.000	4.08–12.18
Addis ababa	1.25	0.287	0.83–1.87	1.43	0.112	0.92–2.21
Dire dawa	5.61	0.000	3.06–10.27	6.89	0.000	3.54–13.41

Backward stepwise model with dichotomous outcome of (0 = no timely initiation; 1 = timely initiation)

*CI* Confidence Intervals

Demographically, the odds of early breastfeeding initiation were more than two and half times higher particularly for mothers residing particularly in regions such as Oromiya (AOR 2.58; 95% CI: 1.84,3.63) and SNNP (AOR 2.75; 95% CI: 1.86,4.05) compared to mothers residing in Tigray. In addition, timely breastfeeding initiation was also significantly associated with wealth index where mothers from wealthier households had 43% higher odds compared to mothers from poorest households (AOR 1.43; 95% CI: 1.07,1.92). Female infants had 17% higher odds of EIBF (AOR 1.17; 95% CI: 1.01, 1.34) compared to male infants. Finally, EIBF was also associated with birth order with recently born children having higher odds of EIBF (AOR 1.44; 95% CI: 1.18, 1.77).

## Discussion

This study showed that overall 7 out of 10 infants were being breastfed within the first hour of birth. Although, this prevalence has significantly improved in the five-year period from 52% in 2011 to 73.4% in 2016, it is still lower when compared to EIBF prevalence in other countries such as Malawi (95.64%), Mozambique (77.74%) and Rwanda (81.51%) [27]. However, the overall prevalence of EIBF in Ethiopia is much higher compared to Central African countries as well as West African countries [27]. This wide variation in the rates of EIBF within Ethiopia and between other African countries is likely due to geographic and cultural differences coupled with economic and health inequalities among different populations.

There was a statistically significant association between EIBF and the following covariates: (i) biomedical factors - type of delivery assistance, (ii) mode of delivery; socio-demographic factors - region, age, gender and birth order of child and (iii) socio-economic factor – wealth index. The predictors of EIBF of 2016 EDHS widely differs from that of the 2011 EDHS [20]. This may be due to several factors not limited to changes in health workforce and infrastructure, changes in nutrition and feeding policies, rural-urban migration, improved education and advancements in medicine and technology [28].

In our study, mothers who delivered with assistance of one or more health professionals had 68% higher odds of timely breastfeeding initiation compared to mothers with no assistance at the time of delivery. This is not surprising as primary health care professionals such as midwives or other trained health professionals would readily inform and assist mothers in the process of achieving timely initiation [29]. This finding is also consistent with findings from within and outside Ethiopia [30, 31].

Similar to existing research in Ethiopia and internationally [16, 17, 32, 33], a strong inverse association was observed between caesarean section and EIBF. Research suggest that mothers with caesarean section fail to initiate timely breastfeeding as they are often hindered by several barriers such as lengthy post-delivery hospital stays, prolonged mother-child separation, delayed skin-to-skin contact and maternal endocrinological diseases [7, 34]. Since delivery through caesarean section is becoming an increasingly common type of delivery, it is imperative to provide services that inform mothers about the importance of EIBF and its wide benefits to their newborn babies and themselves.

Distribution of EIBF rates is significantly different across the regional states in Ethiopia. Mothers from Oromiya, SNNP and Somali had significantly higher odds of EIBF whereas Affar had significantly lower odds of EIBF compared to mothers from Tigray. Other regions such as Harari, Dire Dawa, Gambella also had higher odds of EIBF but may be inconclusive given the low proportion of residents in those regions. This could be due to reasons of better access and availability of health resources in Oromiya, SNNP and Somali compared to Tigray and Afar.

In this study, female infants had higher odds of EIBF compared to male infants. This could potentially be due to the African cultural beliefs that male infants privileged enough to receive prelacteal feeds are accepted by the society as strong and healthy. The practice of prelacteal feeds in male infants is a common practice in African [35, 36] and Asian countries [37, 38].

This study also found a positive association between EIBF and child’s birth order. This is due to the fact that previous breastfeeding experience was positively associated with both intention as well as timely breastfeeding initiation [39]. This positive experience may be due to positive changes in beliefs regarding breastfeeding, where a mother found to benefit from timely breastfeeding initiation may decide to breastfeed a subsequent child in a timely manner. This finding is also consistent with findings from Amibara district of North-Eastern Ethiopia [17].

As expected, mothers from wealthier households had significantly higher odds of EIBF compared to mothers from poorest households. This could be due to several reasons such as better access and availability of health resources and better intellectuality through high quality education. Similar findings have been reported elsewhere [7, 40].

### Policy and practice implications

EIBF rates in Ethiopia have significantly improved in the five-year period possibly as a result of improvement in health workforce, feeding policy, maternal and child health awareness programs. However, this study shows that there are still 3 in 10 infants who are not being breastfed in a timely manner and are not benefitting from the timely initiation, thereby prone to potential health risks. Therefore, a substantial increase in EIBF practice can be achieved by better informing mothers residing regional areas with less access to health services, mothers delivering through caesarean section with less birth term intervals. In addition, primary health care services must also be aimed at mothers from a poorer economic status with adequate resources and counselling about the beneficiary impacts of timely initiation of breastfeeding.

### Strengths and limitations

One of the strengths of this study is that we used data from the 2016 EDHS which is a national survey. Therefore, the study findings have profound implications at person-level, community-level as well as policy-level. However, some local areas represented in the survey had small sample sizes, and thus the results should be interpreted with caution. Since this study is a secondary data analysis of a national survey, other key variables such as traditional beliefs, psycho-social factors, partner’s preference for breastfeeding, in-depth qualitative views of the mothers are not included. This study is based on cross-sectional data and hence it is difficult to demonstrate the cause and effect relationships of the co-variates on timely initiation of breastfeeding and the survey responses may be prone to a recall bias.

## Conclusion

Timely initiation of breastfeeding in Ethiopia is inextricably associated with socio-demographic, bio-medical, and socio-economic factors. Health promotion programs for timely initiation of breastfeeding should be targeted towards mothers residing in the rural Ethiopian communities, those who are more likely to have a caesarean delivery. In addition, primary health care services must also be aimed to better inform mothers from a poorer household by providing adequate resources and counselling about the short and long-term benefits of EIBF to their newborn babies and themselves.

## Abbreviations

AOR: Adjusted odds ratio
BMI: Body mass index
CI: Confidence interval
DHS: Demographic health survey
EDHS: Ethiopian demographic and health survey
EIBF: Early initiation of breastfeeding
GEE: Generalised estimating equation
HSDP: Health sector development program
ICF: Inner city fund
SNNP: Southern nations nationalities and peoples
UNICEF: United nations international children’s fund
WHO: World health organization
